# Date of introduction and epidemiologic patterns of severe acute respiratory syndrome coronavirus 2 (SARS-CoV-2) in Mogadishu, Somalia: estimates from transmission modelling of satellite-based excess mortality data in 2020

**DOI:** 10.12688/wellcomeopenres.17247.2

**Published:** 2022-02-18

**Authors:** Mihaly Koltai, Abdihamid Warsame, Farah Bashiir, Terri Freemantle, Chris Reeve, Chris Williams, Mark Jit, Stefan Flasche, Nicholas G. Davies, CMMID COVID-19 working group, Ahmed Aweis, Mohamed Ahmed, Abdirisak Dalmar, Francesco Checchi

**Affiliations:** 1 London School of Hygiene & Tropical Medicine, London, UK; 2 Somali Disaster Resilience Institute, Mogadishu, Somalia; 3 Satellite Applications Catapult, Didcot, UK

**Keywords:** COVID-19, SARS-CoV-2, transmission model, excess mortality, underascertainment, Somalia, COVID-19 in low-income countries

## Abstract

**Background: **In countries with weak surveillance systems, confirmed coronavirus disease 2019 (COVID-19) deaths are likely to underestimate the pandemic’s death toll. Many countries also have incomplete vital registration systems, hampering excess mortality estimation. Here, we fitted a dynamic transmission model to satellite imagery data of cemeteries in Mogadishu, Somalia during 2020 to estimate the date of introduction and other epidemiologic parameters of the early spread of severe acute respiratory syndrome coronavirus 2 (SARS-CoV-2) in this low-income, crisis-affected setting.

**Methods:** We performed Markov chain Monte Carlo (MCMC) fitting with an age-structured compartmental COVID-19 model to provide median estimates and credible intervals for the date of introduction, the basic reproduction number ( *R _0_*) and the effect of non-pharmaceutical interventions (NPIs) up to August 2020.

**Results:** Under the assumption that excess deaths in Mogadishu March-August 2020 were attributable to SARS-CoV-2 infections, we arrived at median estimates of November-December 2019 for the date of introduction and low *R _0_* estimates (1.4-1.7) reflecting the slow and early rise and long plateau of excess deaths. The date of introduction, the amount of external seeding, the infection fatality rate (IFR) and the effectiveness of NPIs are correlated parameters and not separately identifiable in a narrow range from deaths data. Nevertheless, to obtain introduction dates no earlier than November 2019 a higher population-wide IFR (≥0.7%) had to be assumed than obtained by applying age-specific IFRs from high-income countries to Somalia’s age structure.

**Conclusions:** Model fitting of excess mortality data across a range of plausible values of the IFR and the amount of external seeding suggests an early SARS-CoV-2 introduction event may have occurred in Somalia in November-December 2019. Transmissibility in the first epidemic wave was estimated to be lower than in European settings. Alternatively, there was another, unidentified source of sustained excess mortality in Mogadishu from March to August 2020.

## Introduction

By September 2021, more than 4.7 million people were confirmed to have died from the coronavirus disease 2019 (COVID-19) pandemic caused by the novel severe acute respiratory syndrome coronavirus 2 (SARS-CoV-2). While the cumulative rate of confirmed deaths has exceeded 1-2 per 1000 persons in several European countries, the United States and some of Latin America, it has remained one or even two orders of magnitude lower in most of Africa.

While some of this difference can be potentially explained by a lower infection fatality ratio (IFR) for the entire population due to a lower median age ^1– 3^, evidence suggests that at least critically ill COVID-19 African patients experience higher, not lower mortality than elsewhere ^4^, as plausibly expected due to weaker health infrastructure ^5^. News reports, studies using seroprevalence ^6, 7^, polymerase chain reaction (PCR) testing in morgues ^8^, as well as indirect data sources such as obituaries on social media ^9^ point to substantial under-ascertainment of cases and deaths in low-income countries, potentially ten-fold (suggested by excess mortality data from Egypt ^10^) and in some crisis-ridden regions perhaps even hundred-fold ^11^. While in high income countries confirmed COVID-19 deaths are approximately in line with excess death statistics ^10^, in many African countries there are no reliable mortality statistics, precluding the use of excess death data to infer the true scale of the pandemic.

Additionally, while the first COVID-19 cases in sub-Saharan African countries were identified in late February ^12^ (16th of March in Somalia), there is considerable uncertainty about the true date of introduction, often estimated to be in January 2020 for Western Europe ^13^, or as early as December 2019 according to retrospective PCR on routine patient samples ^14^. For these reasons, alternative data sources such as obituaries ^9^ and satellite imagery ^15^ of cemeteries have been leveraged to estimate the true scale of COVID-19 mortality and its early spread in low- and middle-income countries in Africa and elsewhere.

Burial data derived from cemetery records or from satellite imagery have been used by multiple studies of the COVID-19 pandemic in low-income countries. Analysis of burial records from Ethiopia’s capital, Addis Ababa, showed significant excess mortality in the third quarter of 2020 ^16^. Analysis of mortality records from Jakarta, Indonesia ^17^ showed a rise in excess mortality starting in January-February 2020, more than two months before reported COVID-19 deaths, and an approximately 60% increase in deaths in the first 10 months of 2020 compared to the preceding 5 years. In a study ^15^ of the Aden governorate in Yemen, very high-resolution satellite images of all active cemeteries and Civil Registry office records of deaths were compared, providing validation for the use of satellite imagery. This study found substantial excess mortality in the period April–July 2020, unreported in COVID-19 statistics, with the satellite and registry data showing similar trends, but estimates from satellite imagery approximately 40% lower at the time of peak mortality. The interpolation method from this study to impute graves in the absence of sufficient resolution was also used in the current study.

In the current study we used a dynamic transmission model to analyse a time series of excess deaths in Mogadishu (Somalia) inferred from satellite images of the six main cemeteries in the city ^18^. Our aim was to estimate the probable date of introduction of SARS-CoV-2, as well as the basic reproduction number ( *R _0_*) and the effect of non-pharmaceutical interventions. While we could not verify estimates from satellite imagery with alternative data sources due to the unavailability of death registry data, similar patterns of excess mortality were inferred in other low-income countries based on data from cemeteries ^15– 17^, and the similar study on Yemen ^15^ suggests satellite imagery captures well the trend (if less the absolute numbers) of excess mortality when compared with death notifications to civil registries. While the absolute number of deaths might not be very accurately estimated by satellite imagery it nevertheless remains an important, and in the case of Somalia, to date, the only source to reconstruct the trajectory of the mortality trends of the initial stages of the COVID-19 pandemic.

## Methods

### Ethical approval

This is a modelling study on previously acquired data. There was no primary data acquisition within the framework of the study. For the original underlying data ethical approval was received from the ethics review committee of the London School of Hygiene & Tropical Medicine (REF: 22458) as well as the ethics review committee of the Somali Disaster Resilience Institute (REF: RB-0123).

### Data sources and statistical analysis

All analysis was performed using the free software R (version 3.6.3) used in Rstudio (version 1.3.1093). The analysis code has been deposited on Zenodo ^19^.

Details of the method for inferring excess mortality from satellite images are described in an accompanying article ^18^. Briefly, cemeteries in the Banadir administrative region, which contains Mogadishu, were identified via open-source location data and satellite imagery (OpenStreetMap, Google Earth, GoogleMaps) by a combination of automatic image recognition and manual annotation, in addition to key informant interviews and field visits to identified cemeteries. We identified and analysed six cemeteries (Barakaat 1 and 2, Calamada, Iskool Bolisii, Kahda, Moallim Nuur). We excluded from the analysis five smaller private and family-owned cemeteries estimated to account for less than 20% of all burials because of lack of images and vegetation cover. One cemetery (Calamada) included in the analysis falls outside the limits of Banadir, but largely caters to Mogadishu residents and was therefore included.

In total, 68 archive satellite images from the period February 2017-September 2020 were selected on the criteria that they were cloud-free, of high radiometric quality and with a spatial resolution of 30–40 cm per pixel, were analysed through manual and semi-automated image processing to extract surface area and number of graves. An exhaustive grave count by either of these two methods was possible for 40 out of 68 satellite (58.8%) images. For the remaining images, the number of graves was extrapolated from visible areas or imputed through a generalised additive mixed model of the association between graves and surface area. Results for each image were then interpolated and summed across all cemeteries to yield a single time series of burials for the city.

To compute the baseline (pre-pandemic) crude death rate (CDR), population denominators for Mogadishu (Banadir region) were estimated using the WorldPop project’s database ^20^, using either the 2015 or 2019 estimates, while also adjusting for in- and out-displacement to/from the city ^21^. The two alternative base estimates correspond to a ‘high’ and a ‘low’ scenario with nearly identical trends ( *Extended data,* SI Figure 1 ^22^) and marginally different levels (0.04–0.05 deaths/10.000 person-days) of baseline (i.e. pre-pandemic) CDR.

This level is significantly lower than previous CDR estimates for Somalia ^23^ that were between 0.2–0.6/10.000 person-days. Assuming that the level of under-estimation remains constant, we can scale the crude death rate estimated from our time series up to previous estimates to 0.1–0.4 deaths per 10.000 person-days, using the lower half of the estimates from 23. In terms of modelling transmission dynamics, such scaling of deaths merely shifts the IFR while leaving other parameter estimates unchanged, and hence provides little additional information. We therefore used the observed time series of burials directly for model fitting, without scaling. To isolate excess mortality (which we assumed to be entirely attributable to SARS-CoV-2 infection: see Discussion), we extrapolated pre-2020 burial rates into the pandemic period and subtracted this baseline from the total ( *Extended data*, SI Figure 1 ^22^). Specifically, to calculate excess burials (mortality) we took the daily number of burials in the dataset and subtracted the mean level of daily burials in the four months period 01/07/2019-01/11/2019 (9.3 burials/day). We chose this limited pre-pandemic period as a basis of comparison since burial rates in the preceding period had been likely affected by the drought-triggered crisis 2017-2018 ^23^. The model output of incident deaths was fitted to this baseline-subtracted number of burials per day.

### Transmission model

We used CovidM, an age-stratified dynamic transmission model initially developed to model the spread of COVID-19 and the effect of non-pharmaceutical interventions in the UK ^24, 25^. The model has a susceptible-exposed-infectious-recovered structure with individuals stratified into 5-year age bands. When susceptible individuals are infected they move into an exposed (incubating) compartment (E), becoming either infectious with symptoms (I _c_) following a pre-symptomatic phase (I _p_), or remaining asymptomatic (I _s_) with a lower level of infectiousness (set to 50% as in previous studies ^24, 26^). We used existing age-dependent estimates ^24^ for the proportion of individuals who are symptomatically infected (clinical fraction), as well as for the susceptibility to infection ( *Extended data*, SI Figure 5 ^22^). Both of these estimates are age-dependent, with the clinical fraction 29% in the 0–9y age group and 69% above 70 years, and susceptibility among individuals aged 0–19y half of that among adults. Deaths occur in the model with a gamma-distributed delay ^27, 28^ following the transition from exposed (E) to pre-symptomatic (I _p_) state. To account for the lack of intensive care unit (ICU) beds (the available estimate is around twenty ICU beds in the entire Mogadishu area ^18^), we set the mean delay to 15 days, one week less than the estimate used for high-income countries ^26^, to reflect the fact that severe cases were unlikely to receive adequate treatment. Other parameters of disease progression were fixed to consensus estimates in the literature ( *Extended data*, SI Table 1 ^22^). The model was parameterised with the demographic structure of Somalia ^20^ and the total population of Mogadishu (2.2 million as of mid-2020). Since there is no empirical contact matrix available for Somalia, we used the projected contact matrix ^29^ for its neighbour Ethiopia.

### Estimates on infection fatality ratio

We used existing age-specific IFR estimates ^26^ demonstrating a log-linear relationship between age and the IFR. These estimates are from high-income countries and the IFR is likely to be different in Somalia. To account for this difference and also because the IFR cannot be identified from death data only, we fitted the data with multiple IFR estimates. The estimates were shifted upward to reflect the effect of a weak public health infrastructure. To do so, we took the logit of the original IFR at each age group and increased its value ( *Extended data,* SI Figure 6 ^22^), raising the mean IFR for those 75 or older from the original 11.6% to the range of 26–70%. A possible upward shift of IFR values by age groups is supported by recent findings of substantially higher in-hospital mortality in several African countries ^4^ and by mortality estimates from auxiliary sources and modelling ^30^. The population-average IFR (calculated for a randomly chosen infected person) for Somalia would be 0.15% with direct transferring the original estimates and 0.35%, 0.77%, 1.1% and 1.6%, respectively, with the adjusted values (logit increased by 1, 2, 2.5 and 3, respectively; *Extended data,* SI Figure 6 ^22^).

### Input and fitting parameters

We estimated three epidemiological parameters of the model: the date of introduction into Mogadishu, the basic reproduction number ( *R _0_*) and the effectiveness of the NPIs converting the nominal stringency of non-pharmaceutical interventions (NPIs) into the actual reduction of transmissibility. We held two other parameters, the IFR and the size of the initial seeding event (number of incubating infected individuals (E) that entered the region), fixed and performed the fitting for a range of values. The IFR cannot be determined from death data only, whereas the seed size and the date of introduction are inversely related parameters and not separately identifiable. Therefore, we fitted only the three parameters mentioned, for a range of different IFR levels and seed sizes between 20 and 200 (we tested even larger seed sizes; *Extended data,* SI Figure 11 ^22^). For simplicity we placed the seeding event onto a single day; in our deterministic modelling framework a more gradual introduction does not have a significantly different effect. Initial importations were assumed to be 30 to 70 year-old adults.

The effect of NPIs was accounted for by using the Oxford COVID-19 Government Response Tracker (OxCGRT) ^31^, using the *StringencyIndex* variable for the strength of the NPIs. This estimate is based on mitigation policies announced, which we expect not to be entirely (or even significantly) effective. Since we have no independent data source (such as mobility data) on the actual effect of NPIs in Somalia, we introduced a fitting parameter ( *NPI_scale*) representing the effectiveness of NPIs that converts the nominal stringency into actual contact reductions. We distinguished three periods in the NPIs ( *Extended data,* SI Figure 3 ^22^). In the first, the value of *StringencyIndex* increased abruptly (in three days) from 0 to 41% of its maximum on the 18th of March and stayed above 50% until the 30th of June. From the 1st of July to the 29th of August a number of relaxations followed (second period). In the third period, from the 30th of August the *StringencyIndex* started to increase again and did not decrease until the end of our fitting period. The third period is outside the time window of our model fitting. To minimise over-parameterisation, instead of using the full time series of *StringencyIndex* that would introduce significant additional complexity to model dynamics we only took the mean value of *StringencyIndex* in these three periods (0.59, 0.26 and 0.41; *Extended data,* SI Figure 3 ^22^), and implemented the effect of NPIs by reducing transmission coefficients for all age groups by the product of the mean value *StringencyIndex* per period and the NPI effectiveness ( *NPI_scale*). For example, if *NPI_scale*=0.5, then the actual reduction in transmission was 29.5% (since it is the product StringencyIndex* *NPI_scale*= 0.59*0.5) in the first period when stringency was 0.59, and 13% ( StringencyIndex* *NPI_scale*=0.26*0.5) in the second period.

### Time window of fitting

All fits presented in the main text were done with data within the time window 02/03/2020 to 24/08/2020, excluding the first smaller spike of deaths in January-February, as well as the late spike in September. We removed the January–February spike in excess burials to avoid any confounding from the two continuous weeks of reported cholera deaths in Banadir ^31^ that coincided with this period (see Discussion). Moreover, this early spike of deaths is in general inconsistent with a gradually rising epidemic curve from the beginning of March. Including the deaths in January–February leads to even earlier estimates for the date of introduction, but poorer fits ( *Extended data,* SI Figure 10 ^22^), as the epidemiological model cannot capture this early non-monotonic dynamics.

### Fitting procedure

To estimate the unknown parameters, we fitted the CovidM model to the time series of excess deaths using a Monte Carlo Markov chain (MCMC) algorithm, minimising the log-likelihood of incident deaths (assumed to be Poisson-distributed). We introduced informative prior assumptions for the date of introduction (normal distribution with mean: 01/03/2020, standard deviation: 20 days) and *R _0_* (truncated normal distribution with mean=3, standard deviation=1, bounded at 1 and 5), and an uninformative uniform distribution for the NPI scaling factor (U(0,1)). We used a differential evolution MCMC algorithm with 10 chains, with a burn-in of at least 500 iterations followed by at least 2000 samples.

## Results

**Figure 1.**
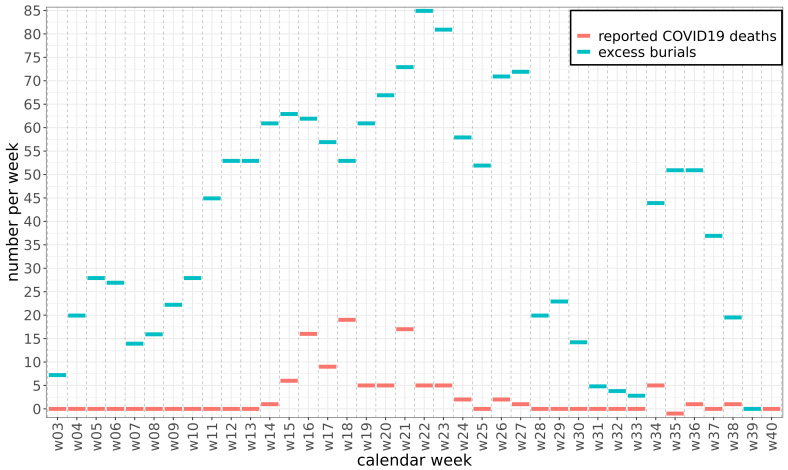
Excess burials and reported coronavirus disease 2019 (COVID-19) deaths. Weekly burials above the pre-pandemic baseline (excess burials) in Mogadishu compared to reported COVID-19 deaths in Somalia.

Satellite imagery of the six main cemeteries in Mogadishu showed a first spike in the number of burials in late January and February, followed by a more sustained rise from early March ( Figure 1). The weekly number of excess burials rose to approximately 60 in April and to a peak of 85 in mid-June, falling back to values near zero only in August ^32^.

### Estimates for date of introduction and initial spread ( *R _0_*)

**Figure 2.**
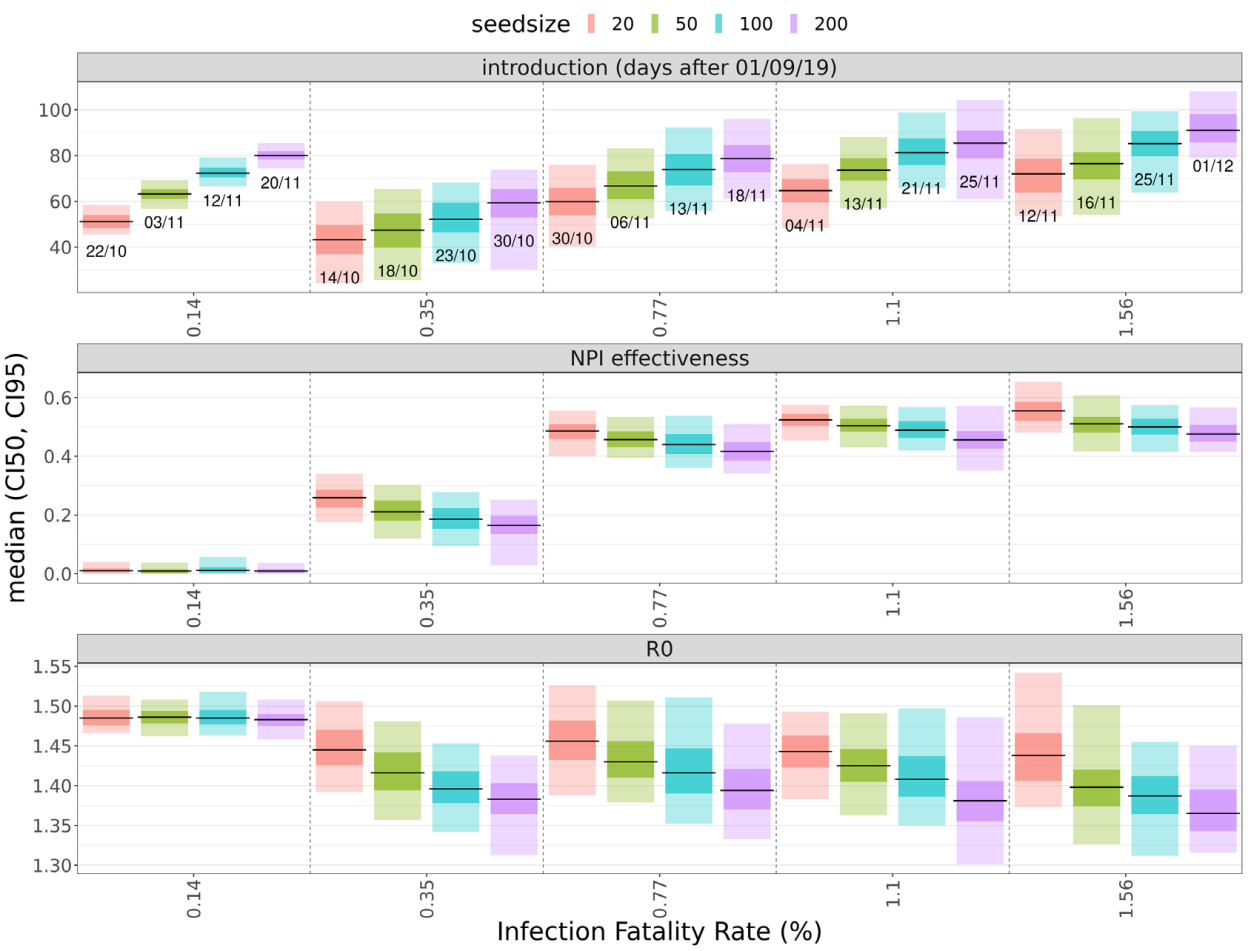
Estimates of fitting parameters. Median values and credible intervals for the fitting parameters (introduction date, NPI effectiveness [NPI_scale], R _0_) and quality of fits at different assumed values of the infection fatality rate (x-axis) and seed size (colors). In the top panel, labels below the lines show median estimates of the date of introduction (dd/mm, all dates are in 2019). Shaded areas around the median (black) are 50% (darker) and 95% credible intervals.

**Figure 3.**
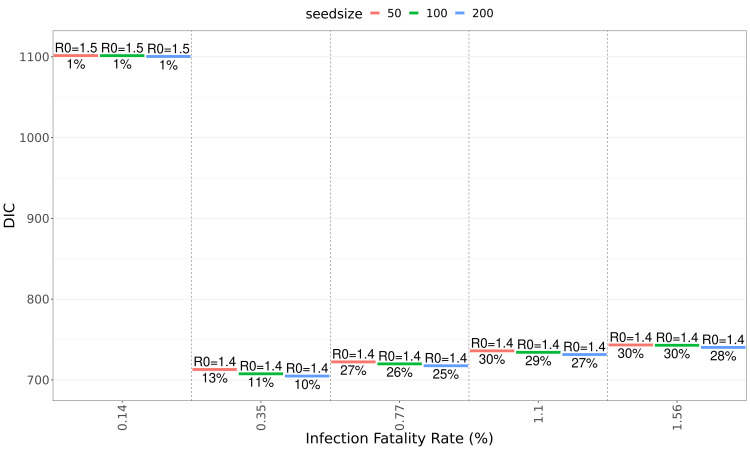
Quality of fits for different infection fatality ratios and seed sizes. Goodness of fit as measured by DIC (deviance information criterion) at different values of seed size and population-wide IFR (infection fatality rate). Labels above the coloured lines show median estimates for R _0_, labels below show the NPI-induced reduction in transmissibility (during the first NPI period).

The slow rise in deaths from March to mid-June and the long plateau lasting until late July results in *R _0_* estimates substantially lower than those for Wuhan ^33^ and European countries ^25^ in the initial phase of the pandemic. Fitting our data with IFR values between 0.15% and 1.6% and seed sizes from 20 to 200 resulted in median *R _0_* estimates of 1.4-1.5 ( Figure 2– Figure 3, Table 1). Fit quality as expressed by DIC (deviance information criterion) values are similar for population-wide IFRs between 0.36% and 1.6% ( Figure 3), but much poorer when using the high-income country-specific base assumption of 0.15%.

**Table 1.** Parameter estimates. Mean values and 95% credible intervals of the three fitting parameters, for different values of the infection fatality rate (IFR) and the seed size. NPI=non-pharmaceutical interventions.

Parameter description	IFR=0.77%	IFR=1.1%	IFR=1.6%	seed size
date of introduction (dd/mm, all dates 2019)	*30/10 (11/10, 16/11)*	*04/11 (19/10,16/11)*	*12/11 (24/10,01/12)*	**20**
	*06/11 (23/10, 23/11)*	*13/11 (28/10,28/11)*	*16/11 (25/10,06/12)*	**50**
	*13/11 (26/10, 02/12)*	*21/11 (05/11,08/12)*	*25/11 (03/11,09/12)*	**100**
	*18/11 (01/11, 05/12)*	*25/11 (31/10,14/12)*	*01/12 (19/11,18/12)*	**200**
*R _0_*	*1.46 (1.39,1.53)*	*1.44 (1.38,1.49)*	*1.44 (1.37,1.54)*	**20**
	*1.43 (1.38,1.51)*	*1.43 (1.36,1.49)*	*1.4 (1.33,1.5)*	**50**
	*1.42 (1.35,1.51)*	*1.41 (1.35,1.5)*	*1.39 (1.31,1.46)*	**100**
	*1.39 (1.33,1.48)*	*1.38 (1.3,1.49)*	*1.36 (1.32,1.45)*	**200**
*NPI_scale (NPI effectiveness)*	*0.49 (0.4,0.56)*	*0.52 (0.45,0.57)*	*0.56 (0.48,0.65)*	**20**
	*0.46 (0.4,0.54)*	*0.5 (0.43,0.57)*	*0.51 (0.42,0.61)*	**50**
	*0.44 (0.36,0.54)*	*0.49 (0.42,0.57)*	*0.5 (0.42,0.57)*	**100**
	*0.42 (0.34,0.51)*	*0.46 (0.35,0.57)*	*0.48 (0.42,0.57)*	**200**

**Figure 4.**
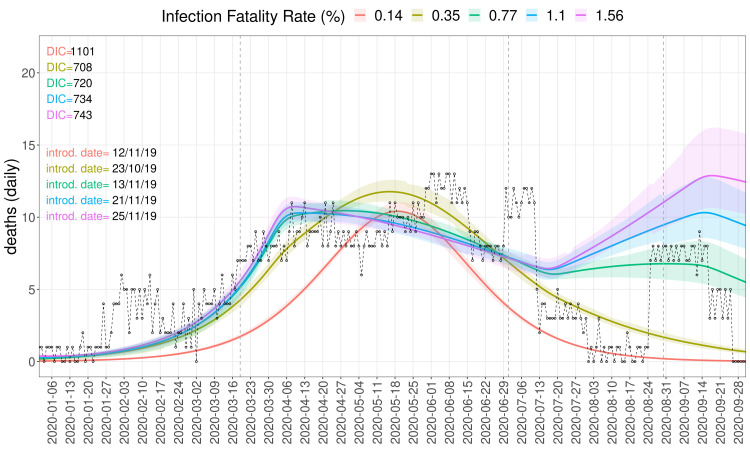
Burial data and simulated deaths. Dynamics generated by sampling the posterior distributions of fitting parameters, at a seed size of 100 and four IFR values from 0.14% to 1.56%. Different colours correspond to separate fits with different assumed IFR values, with the solid lines corresponding to the median of the posterior distribution and the shaded regions the 95% confidence intervals. The dashed black line and circles show the daily number of excess burials. The period from 2 March to 24 August was used for fitting.

Using population-wide IFR values below 0.5% leads to implausibly early date of introduction estimates before November 2019 ( Figure 3). The lowest IFR value also leads to a higher R _0_ estimate, as a larger wave of infections is required to fit the number of deaths in the data, however, this fit is unable to account for the dynamics of deaths ( Figure 4). With progressively higher assumed population-wide IFRs, the median date of introduction estimate gradually moves to mid- and late November 2019, or early December when assuming a large (200 introductions) seeding event. Even larger seeding events of 500 or 1000 introductions shift the median date of introduction estimates to mid-December for the highest IFR values ( *Extended data,* SI Figure 11 ^22^). In other words, our excess mortality time series can be fitted either with an early introduction date or a very large amount of external seeding at a later date. However, even in the case of up to 1000 external introductions, the date of introduction is still mid-December 2019, earlier than most previous estimates.

### Estimates for the effect of NPIs

**Figure 5.**
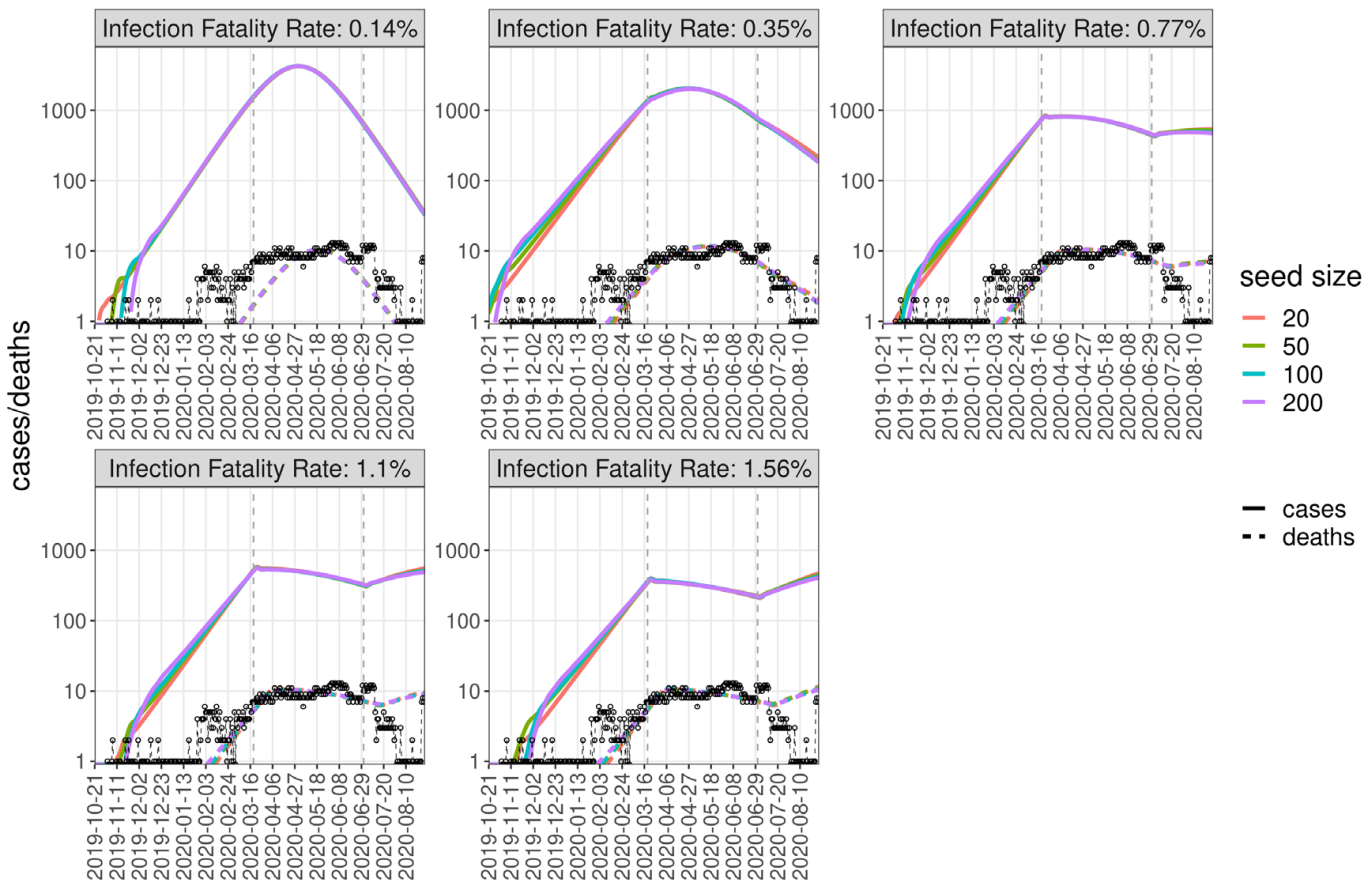
Simulated dynamics of cases and deaths. Simulated dynamics of cases (solid lines) and deaths (dashed, coloured) for different seed sizes (colours) and IFR values (subplots) using the mean values of fitted parameters, compared to daily number of excess burials (black dashed line and circles).

Estimates for the effectiveness of NPIs show positive correlation with the population-average IFR and (weaker) negative correlation with the seed size ( Figure 2). For the first NPI period, when NPIs were the most stringent (19/March to 30/June), we obtained median estimates for the NPI-caused transmissibility reductions between 25 and 30% for the IFR values ( Figure 3) 0.77% and above. These values are lower than NPI-induced reductions of contact rates in high income countries ^34^, however they do have an effect on the growth of cases for the fits with IFR values of 0.77% and above, as they break the exponential growth of cases, resulting in the long plateau of deaths observed in our burial data ( Figure 4– Figure 5). This breakpoint in the dynamics of infections due to the stronger effect of NPIs for the fits with IFR=0.77% and above also shifts the date of introduction to mid- to late November 2019 ( Figure 2 and Figure 4), whereas the fits for lower IFRs yield lower estimates for NPI effectiveness but implausibly early introduction dates (October 2019).

### Uncertainty in estimates due to parameter correlations

There are strong positive correlations between the three fitting parameters ( *Extended data,* SI Figure 8 ^22^), nevertheless 50% and 95% credible intervals for all three are relatively narrow ( Figure 2). A higher assumed IFR value and a larger seed size both shift the date of introduction to later dates, and larger assumed seed sizes also lower estimates for the basic reproduction number ( Figure 2– Figure 3). The quality of fits in terms of DIC values are essentially the same for the IFR input values of 0.35% and above ( Figure 3). Seed sizes larger than 200 do not improve the fit quality further ( *Extended data,* SI Figure 11 ^22^) while being less likely themselves.

### Estimates using only the initial phase of excess deaths

The early estimates of the date of introduction are not only due to the early appearance of excess deaths in our data, but also due the low R _0_ estimates, which are in turn affected by the long plateau of deaths over the summer of 2020 after a first exponential phase. Estimates of the number of burials from satellite imagery in Yemen ^15^ indicated differential underestimation of deaths (compared to death notifications to the civil registry) at the peak of excess mortality, although still correctly reflecting the general trend of excess mortality. Therefore, it is possible that the real peak of deaths was higher and the excess mortality curve had a sharper exponential rise, but the number of burials underestimates excess deaths during the peak period of the pandemic, perhaps because of out-migration from the city (as observed in India ^35^) during the pandemic or opening of new burial sites not included in our satellite data. While field visits and interviews ^18^ did not identify new burial sites and we therefore cannot ascertain the veracity of this hypothesis, we approximated it by re-fitting the model to the pre-peak period up to 13 April only. This led to a shift in the date of introduction estimates to the period of mid-November to mid-December for seed sizes of 20 to 100 ( *Extended data,* SI Figure 9 ^22^), with median *R _0_* estimates rising to 1.4–1.7. Date of introduction estimates in this case are no earlier than mid-November even in the case of a seed size of 20.

### Deaths from political violence in 2020

Since Somalia is heavily affected by political violence, including armed conflicts and terror attacks ^36^, we also investigated if a concurrent rise in violent deaths could explain the sustained rise in burials observed March-August 2020. We analysed the number of fatalities due to political violence documented for Somalia in the Armed Conflict Location & Event Data Project (ACLED) database for the years 2018, 2019 and 2020. Compared to 2018-2019, for the year of 2020 we found no increase, but rather a reduction, in the number of fatalities due to political violence in the Banadir region ( *Extended data,* SI Figure 4 ^22^). While there was one major terrorist attack claiming 85 lives on the 28th December 2019, this was followed by a long period of deaths below the level of the previous year, followed by two major incidents (20 and 26 deaths, respectively) in August 2020. In the period from February to July, when the daily burial rate doubled from its baseline, there was no increase of fatalities due to political violence, therefore we find no evidence supporting the hypothesis that the rise in burials could be due to this exogenous factor.

## Discussion

Fitting excess mortality in Mogadishu from the beginning of March by a validated SARS-CoV-2 age-structured compartmental model ^24, 26^ we arrived at date of introduction estimates from mid-November to early December 2019, at least one month earlier than previous estimates ^37^. The estimate shifts to mid-December only by assuming one thousand or more external introductions ( *Extended data,* SI Figure 11 ^22^). Additionally, our estimates of the basic reproduction number between 1.4 and 1.7 are also lower than previous estimates ^38^. These two findings are not only due to the early appearance of excess mortality, but also the slow rise of deaths and their sustained plateau from April to July, leading to low *R _0_* estimates and a consequent dating-back of the introduction date to early time points. While the epidemiological model can fit the deaths data relatively well ( Figure 4– Figure 5), the introduction dates of mid-November to early December 2019 are surprising based on the current understanding of the early phase of the COVID-19 pandemic, usually dating the introduction of the pathogen to Africa to January 2020 ^12, 39^.

This study has several limitations. Our model fitting of excess deaths is predicated on the strong assumption that the unexplained rise in burials from March 2020 was due to deaths caused by SARS-CoV-2 infection. We investigated a number of alternative hypotheses other than COVID-19 that could explain the observed excess mortality.

There was an ongoing cholera epidemic in Somalia following floods in 2017 ^31^, resulting in 19 confirmed deaths in Banadir (mostly children) from January to October 2020. In the four weeks of 20 January to 06 February, approximately coinciding with the first transient increase of excess burials in our dataset, there were four confirmed cholera deaths in the whole of Somalia, after a preceding period of no reported deaths. In the period from 16 February to 12 April there were eight further cholera deaths reported Somalia-wide, and another 12 deaths in June-July. These numbers are much lower than the observed total increase of burials between February and October 2020: approximately 1500 excess burials were directly identified from satellite imagery and we estimated total excess deaths in Banadir to be between four and twelve thousand. While some underestimation of cholera-related deaths is possible, due to its well-identifiable pathology we consider it unlikely that a major cholera outbreak was almost entirely missed and could explain a substantial proportion of the excess mortality.

There are two, non-exclusive ways to interpret these findings. On the one hand, given uncertainties about the earliest phase of the pandemic in Wuhan ^40^, it is possible that SARS-CoV-2 was imported to Mogadishu at an earlier date than most consensus estimates. Mogadishu, the only international airport in Somalia, received flights with over thirty thousand seats in total per month in the period before the COVID-19 pandemic ^39^. The country has connecting flights with multiple countries (UAE, Turkey, Kenya, Ethiopia, Qatar) that have several daily flights with China and in two cases (Turkey, UAE) with Wuhan. Moreover, news articles reported at least 34 Somali students in Wuhan, with the entire Somali diaspora likely to be larger, as trade and general economic relations between China and Somalia have been expanding in the last two decades, resulting in a growing Somali (and other African) diaspora in China ^41^. Larger neighbouring countries Kenya and Ethiopia have far more extensive trade ^42^ and travel flows with China, making indirect importation to Somalia possible, but implying that earlier introduction dates could have happened for those countries as well. SARS-CoV-2 positive routine samples from mid-December 2019 were also found in Italy ^14^, France ^43^ and the United States ^44^, suggesting the pathogen was circulating in small numbers by the end of 2019 outside of China, but resulting in excess mortality only from Spring 2020. Phylogenetic analysis ^45^ suggests that a progenitor of the SARS-CoV-2 variant first identified in Wuhan might have been spreading outside of China before the known beginning of the city’s outbreak.

In the compartmental-deterministic framework we used, superspreading events can only be incorporated as static model inputs (i.e., an injection of cases into the model), although the large seed sizes used as input parameters can be interpreted as proxies for early superspreading events that followed smaller seeding events. In Somali society, superspreading events such as large funerals or marriages may be more likely than in Europe, such that the importation of even a few seed cases in late 2019 might have resulted in extensive early propagation.

If some of the excess mortality observed in our dataset was due to causes other than COVID-19, a more sharply rising epidemic curve might be hidden within the curve of all excess deaths, which, if disentangled, would lead to a higher *R _0_* estimate and therefore a later date of introduction. We tried to approximate this potential effect by an alternative fit of only the first, exponential phase of the excess mortality curve, but not the ensuing long plateau which might have been due to the indirect effects of the pandemic. This alternative fit resulted in arguably more plausible estimates of the date of introduction, up to mid-December 2019. Conversely, if a very early introduction did occur, leading to a rise in deaths from early March 2020, behavioural adaptation by the general population might have reduced contact rates, resulting in a lower *R _0_*. During 2020 Mogadishu was not reported to be affected by large-scale armed conflict, influx of displaced people or food insecurity, as in previous phases of the protracted crisis in Somalia. Differential under-ascertainment of burials over time in the satellite imagery analysis may therefore provide a more plausible explanation. Burials in the deceased’s village of origin outside the capital and a potential decrease in the number of these burials (and thereby an increase of burials within city limits) due to mobility restrictions could have also played a role. It is also plausible that some of the excess mortality, especially its long plateau over the summer of 2020, was due to the NPIs themselves and other socio-economic disruptions due to the pandemic, though they cannot explain the early rise in excess burials predating NPIs.

Our best fits were obtained at IFR values higher than if age-specific IFRs were identical to consensus estimates from high-income countries. With the above qualifications in mind, this finding can be interpreted as supporting a higher IFR than expected from age demographics only, which could be due to untreated comorbidities and limited access to treatment. Raising the assumed population-average IFR value from 0.35% to 1.6% shifted the date of introduction estimates by approximately one month ( Figure 2 and Figure 3). Even higher IFR values than 1.6 would require the IFR for those older than 75 to reach unrealistically high levels (over 70%). However, while in order to avoid over-parameterisation we shifted (the logit of) age-specific IFR values uniformly, it is possible that this shift was in reality differential and more pronounced for the larger younger age groups ^30^. This could raise the population-average IFR above 1.6%, potentially shifting the estimated date of introduction to a later date.

The *R _0_* estimates between 1.4-1.7 are substantially lower than consensus estimates for SARS-CoV-2 for China ^46, 47^, Europe ^25, 48^ or the United States ^49^. There is no empirical contact matrix or real-time mobility data available for Somalia, it is therefore possible that contact structures are in reality somewhat different from the projected contact matrix ^29^ for its neighbour (Ethiopia) that we used for model fitting, contributing to a lower reproduction number. Another possibility is that susceptibility to infection in younger individuals ( *Extended data,* SI Figure 5 ^22^) is lower than estimates inferred for middle and high-income countries ^24^, i.e., that the lower median age in Somalia reduced the *R _0_* further. Relatively high ventilation of houses and proportion of time spent outdoors due to warm weather may also have reduced transmissibility. Other factors such as cross-immunity have also been proposed ^50^.

Finally, we note that our model fitting resulted in attack rates between 15–50% ( *Extended data,* SI Figure 12 ^22^, which would have left a large pool of susceptibles for a second wave to develop if the reproduction number increased due to introduction of more contagious new variants in late 2020. Indeed, a reportedly sharp pandemic wave was observed in Somalia ^51^ from late February to May 2021.

In summary, our analysis was based on the assumption that the rise in excess mortality observed via satellite imagery of cemeteries in Mogadishu starting early 2020 was due to deaths caused by SARS-CoV-2 infection. Under this assumption, model fitting of the time series of deaths suggests that SARS-CoV-2 could have been introduced to Somalia’s capital substantially earlier than previously thought and had a basic reproduction number (R _0_) lower than consensus estimates from middle- and high-income countries. To obtain date of introduction estimates no earlier than mid-November 2019 we had to assume population-wide IFR values of 0.7% or above, substantially above the value obtained by applying age-specific IFR estimates from high income countries to Somalia’s age structure (0.15%). Even assuming thousands of external introductions of SARS-CoV-2 the median date of introduction estimate is shifted to mid-December only ( *Extended data,* SI Figure 11 ^22^). Alternatively, there was another source of sustained excess mortality in Mogadishu from March to August 2020. If these excess deaths were indeed due to SARS-CoV-2 infections, this raises several questions about the pathogen’s introduction and the true burden of the pandemic in Somalia and countries in the region. Further investigation of mortality trends and SARS-CoV-2 epidemiology in Somalia and other low-income countries is warranted to paint a more conclusive picture, and help to better predict future waves of the pandemic in these settings.

## Data availability

### Underlying data

Zenodo: Source data for "Date of introduction and epidemiologic patterns of SARS-CoV-2 in Mogadishu, Somalia: estimates from transmission modelling of satellite-based excess mortality data in 2020". https://doi.org/10.5281/zenodo.5534769 ^32^.

This project contains the following underlying data:

- 2018-04-23-2021-04-28-Somalia.csv (ACLED data on political violence)
- df_compare_report_satell.csv (satellite data on number of burials compared to reported deaths)

The .rds files below are from the MCMC fitting, dates showing the time window of fitting, the value after “ifr_increm” the increment to the logit of the IFR curve, and the value after “seedsize” the number of external introductions:

- fits_death_2020-03-02_2020-08-02_ifr_increm0_seedsize100.rds
- fits_death_2020-03-02_2020-08-02_ifr_increm0_seedsize20.rds
- fits_death_2020-03-02_2020-08-02_ifr_increm0_seedsize200.rds
- fits_death_2020-03-02_2020-08-02_ifr_increm0_seedsize50.rds
- fits_death_2020-03-02_2020-08-02_ifr_increm1_seedsize100.rds
- fits_death_2020-03-02_2020-08-02_ifr_increm1_seedsize20.rds
- fits_death_2020-03-02_2020-08-02_ifr_increm1_seedsize200.rds
- fits_death_2020-03-02_2020-08-02_ifr_increm1_seedsize50.rds
- fits_death_2020-03-02_2020-08-02_ifr_increm2.5_seedsize100.rds
- fits_death_2020-03-02_2020-08-02_ifr_increm2.5_seedsize20.rds
- fits_death_2020-03-02_2020-08-02_ifr_increm2.5_seedsize200.rds
- fits_death_2020-03-02_2020-08-02_ifr_increm2.5_seedsize50.rds
- fits_death_2020-03-02_2020-08-02_ifr_increm2_seedsize100.rds
- fits_death_2020-03-02_2020-08-02_ifr_increm2_seedsize20.rds
- fits_death_2020-03-02_2020-08-02_ifr_increm2_seedsize200.rds
- fits_death_2020-03-02_2020-08-02_ifr_increm2_seedsize50.rds
- fits_death_2020-03-02_2020-08-02_ifr_increm3_seedsize100.rds
- fits_death_2020-03-02_2020-08-02_ifr_increm3_seedsize20.rds
- fits_death_2020-03-02_2020-08-02_ifr_increm3_seedsize200.rds
- fits_death_2020-03-02_2020-08-02_ifr_increm3_seedsize50.rds
- IFR_by_age_imperial.csv (IFR estimates from 3 Imperial study)
- IFR_estimates_Sandmann2021.csv (IFR estimates from 26)
- out_bdr_daily_estimates.csv (Processed satellite data on number of burials)
- suscept_clinfract_posteriors_davies2010.csv (estimates on clinical fraction and susceptibility to infection from 24

### Extended data

Zenodo: Extended data for "Date of introduction and epidemiologic patterns of SARS-CoV-2 in Mogadishu, Somalia: estimates from transmission modelling of satellite-based excess mortality data in 2020". https://doi.org/10.5281/zenodo.5525349 ^22^.

This project contains the following extended data:

- SI.pdf (Supplementary material for “Date of introduction and epidemiologic patterns ofSARS-CoV-2 in Mogadishu, Somalia: estimates from transmission modelling of satellite-based excess mortality data in 2020”)
- SI_Fig1.tiff (SI Figure 1. Burial rate per 10.000 person-days inferred from satellite imagery by interpolating between data points provided by satellite images (for details of methods see the accompanying paper ^18^). The color shading represents estimates with low and high population denominators.)
- SI_Fig2.tiff (SI Figure 2. A. Burials by cemetery. Barakaat 1 cemetery was mostly filled up by January 2020 and replaced by its extension Barakaat 2. B. Dates for which satellite imagery was acquired.)
- SI_Fig3.tiff (SI Figure 3. ‘ *StringencyIndex*’ from the Oxford COVID-19 Government Response Tracker database. We divided the months of 2020 from March into four periods (separated by solid horizontal lines) and took the average of each. The fourth period is outside the time window of model fitting. The values are equal to the relative reduction in contacts (transmissibility) if the effectiveness of the NPIs ( *NPI_scale*) was 1 (100%).)
- SI_Fig4.tiff (SI Figure 4. Weekly fatalities due to political violence in the Banadir region (from ACLED database) compared to the number of burials, going back to January 2019. The infrequency of satellite imagery analysed before 2020 does not enable identification of acute peaks in burials that would follow a mass casualty incident.)
- SI_Fig5.tiff (SI Figure 5. Clinical fraction and susceptibility by age group. Values from the literature ^24^ (green) were approximated by a piecewise linear function (red) made up of 3 sections (minimum, maximum and a line connecting them). The linearly approximated values were used as model parameters to minimise model complexity.)
- SI_Fig6.tiff (SI Figure 6. IFR estimates by age groups, using estimates from 26. Adjusted curves were calculated by taking the logit of the age-specific IFRs and adding the values 1, 2, 3.)
- SI_Fig7.tiff (SI Figure 7. Dynamic fits with all seed sizes and IFR estimates, fitting the period of 2 March to 24 August 2020. Labels show DIC values and median estimates for the date of introduction for the five different population-average IFR values that we tested.)
- SI_Fig8.tiff (SI Figure 8. Posterior distributions of the three fitting parameters (R0, introduction date, NPI effectiveness (npi_scale)) generated by MCMC fitting, showing correlations between the parameters.)
- SI_Fig9.tiff (SI Figure 9. Model fitting restricted of the period 23 February to 13 April with four different seed sizes.)
- SI_Fig10.tiff (SI Figure 10. Model fitting of the period 23 February to 24 August 2020. Labels show DIC values and median estimates for the date of introduction by population-average IFR values.)
- SI_Fig11.tiff (SI Figure 11. Model fitting of the period from 02 March to 24 August with seed sizes up to 1000)
- SI_Fig12.tiff (SI Figure 12: Epidemic size for different fits. Cumulative attack rates for different seed sizes and IFR values, fitting the period 02/03/2020-24/08/2020.) Different IFR values lead to different estimates of R _0_ and NPI_scale, resulting in different herd immunity thresholds and attack rates.

Data are available under the terms of the Creative Commons Attribution 4.0 International license (CC-BY 4.0).

### Analysis code

The code for model fitting and producing the figures is available at: https://github.com/mbkoltai/covid_lmic_model/tree/v1.0

Archived source code at time of publication: https://doi.org/10.5281/zenodo.5534763 ^19^.

License: MIT
