# Supplementary material for: Date of introduction and epidemiologic patterns of severe acute respiratory syndrome coronavirus 2 (SARS-CoV-2) in Mogadishu, Somalia: estimates from transmission modelling of satellite-based excess mortality data in 2020
Source: Wellcome Open Res. 2022 Feb 18;6:255. Originally published 2021 Oct 6. [Version 2] doi: 10.12688/wellcomeopenres.17247.2 (PMC8902262; doi:10.12688/wellcomeopenres.17247.2)
Supplement: Peer review history

# Peer review: Reviewer response for version 2

**DOI:** 10.21956/wellcomeopenres.19622.r48813

**Published:** 2022-03-07

**Authors:** Bhaswar Ghosh

**Affiliations:** 1 International Institute of Information Technology, Hyderabad, India

The authors present a modeling study regarding COVID-19 spread in Somalia. The study specifically uses death burial data to calculate the reproduction number R _0_. Some of the concerns I expressed, are being addressed adequately.

1. They have used only death burial data to calculate the reproduction number since the daily infection data is limited.
2. They have provided an explanation of systematic change in R _0_ with IFR.
3. They provided a detailed description of the figure 4 regrading the lines with different colors.
4. They have provided an explanation of possible mismatch between the simulation and data for figure 5.

I appreciate the authors for addressing the issues and providing explanation in the main text. I would recommend the article for an approval of indexing.

Is the work clearly and accurately presented and does it cite the current literature?

Yes

If applicable, is the statistical analysis and its interpretation appropriate?

Yes

Are all the source data underlying the results available to ensure full reproducibility?

Yes

Is the study design appropriate and is the work technically sound?

Yes

Are the conclusions drawn adequately supported by the results?

Yes

Are sufficient details of methods and analysis provided to allow replication by others?

Yes

Reviewer Expertise:

Computational Biology, Dynamical models, Biophysics

I confirm that I have read this submission and believe that I have an appropriate level of expertise to confirm that it is of an acceptable scientific standard.

# Peer review: Reviewer response for version 1

**DOI:** 10.21956/wellcomeopenres.19063.r47064

**Published:** 2021-12-20

**Authors:** Bhaswar Ghosh

**Affiliations:** 1 International Institute of Information Technology, Hyderabad, India

The article presents a method to estimate the infection rate and various other parameters based on the death data from satellite images of burial grounds in Somalia. The technique provides a quite important method to estimate the parameters from a different data source. Since the reported case numbers and deaths are heavily underestimated in many poor and middle-income countries, such alternative strategies would be highly pertinent for the current situation. I have a few concerns about the methods in the study.

1. The authors used only the death data to fit the epidemic model. This may underestimate the values of the R _0_, since R _0_ is the actual infection spreading rate. They may have used the reported case numbers also for simultaneously fitting both case numbers and deaths.
2. In Figure 2, a systematic reduction in R _0_ value is observed with assumed fatality rate. They may provide some explanation for this either in the results section or in the discussion.
3. In Figure 4, it seems that the other solid lines (blue, cyan, green) are some estimates of the death rate from the data. This is not mentioned in the figure legends. The authors should mention the description of those lines in the legends to make it clear. In addition, there is a significant increase in death at 2020-8-03 which is not captured by the model, possibly due to the assumption of fixed time-independent parameter values.
4. In Figure 5, it is not clearly mentioned how the simulated curves change discontinuously at 2020-6-29, as it is mentioned that parameter values remain fixed at the mean estimated values.
5. In the methods section (Transmission model), the authors mentioned ‘ *Both of these estimates are age-dependent, with the clinical fraction 29% in the 0-9y age group and 69% above 70 years, and susceptibility among individuals aged 0-19y half of that among adults*'. It is not clear what is the basis for this assumption or it is taken from any other study.

Is the work clearly and accurately presented and does it cite the current literature?

Yes

If applicable, is the statistical analysis and its interpretation appropriate?

Yes

Are all the source data underlying the results available to ensure full reproducibility?

Yes

Is the study design appropriate and is the work technically sound?

Yes

Are the conclusions drawn adequately supported by the results?

Yes

Are sufficient details of methods and analysis provided to allow replication by others?

Yes

Reviewer Expertise:

Computational Biology, Dynamical models, Biophysics

I confirm that I have read this submission and believe that I have an appropriate level of expertise to confirm that it is of an acceptable scientific standard, however I have significant reservations, as outlined above.

# Response

**Published:** 2022-02-15

**Authors:** Mihaly Koltai

**Affiliations:** London School of Hygiene & Tropical Medicine, UK

Dear Dr. Ghosh,

 We thank you for your valuable comments concerning our manuscript on estimating the date of introduction of SARS-CoV-2 in Mogadishu (Somalia) based on satellite imagery of cemeteries. Please find our responses to the points raised below.

 1) It is the case that we only fitted our transmission model to excess mortality data and not case numbers to derive the R _0_ estimate. We would argue that this was the correct and indeed only way to proceed for two reasons.

 First, the transmission model we used (the COVIDM model) transforms infections to deaths using a delay distribution taken from the literature (a gamma distribution with a mean of 15 days). To the extent that there is a delay and a smoothing effect from infections to deaths this is captured by applying this delay distribution to the simulated infections.

 Second, using reported case numbers from Somalia was not a workable option in our case as reported cases only exceeded 10 cases on the 10th of April 2020, well after the rise in burials seen in our data, which started in late February. Therefore, fitting reported case numbers is inconsistent with our dataset and could not be used as a source of comparison, since the data of our interest, i.e.. excess mortality rising from late February is outside the earliest time point of reported cases.

 2) We included an explanation on this in Results. Briefly, since we are fitting to deaths data only, the IFR is not a separately identifiable parameter and therefore we had to run separate fits with different assumed IFR values. When using the lowest assumed IFR (0.14%, see also SI Fig 6; the value was generated by applying age-specific IFR estimates from the literature to Somalia's age structure) this leads to higher R _0_ estimates, as a larger number of infections need to be generated to be able to fit at all the number of deaths in the data. However we note that a) this fit at IFR=0.14% is of poor quality (see Figure 4) b) above this value of IFR the R _0_ does not substantially change with the IFR, instead there is an inverse relationship with the seed size, as a larger initial number of infections and a larger R _0_ are exchangeable (within a certain range).

 3) The solid lines in Fig 4 are generated by sampling from the posterior distributions obtained from MCMC fitting at different assumed values of the IFR. As noted in the previous point the IFR cannot be separately identified when relying on deaths data only, therefore different values had to be assumed and the MCMC fitting performed at these different input (IFR) values. The five solid lines correspond to the medians and the CI95 of the posterior distributions. We added this information to the figure legend.

 The model does not fit well the increase in deaths from the 24th of August because we restricted the time window of model fitting to the period from 02/03/2020 to 24/08/2020. This is because our aim was the estimation of the date of introduction from the early rise of excess deaths. It is possible that the spike in deaths from the 24th of August could be fitted better if we increased the resolution of the 'StringencyIndex' from the Oxford COVID-19 Government Response Tracker as stringency started to fall in early August. We decided to rather introduce the stringency index with a lower resolution (four periods with fixed values) to minimise the number of exogenously introduced parameters.

 4) The values for the stringency of interventions, which are introduced as scaling terms for the contact rates are fixed **within** the four periods that we aggregated the time series of 'StringencyIndex' into, however they change **between** these periods (SI Figure 3). Specifically the averaged stringency index falls from 0.6 to 0.26 on the 1st of July, causing the spike in infections (and later in deaths) for the three fits with the highest assumed IFR values.

 5) The source for the age-specific curve of susceptibility and the clinical fraction is given in the previous sentence, it is reference [24] (Davies NG, Klepac P, Liu Y, et al.: Age-dependent effects in the transmission and control of COVID-19 epidemics. Nat Med. 2020; 26(8): 1205–11). The age-dependent susceptibility and clinical fraction curves are shown in SI Fig 5.

 We hope that we have answered all your comments and you will be able to approve the article. We are happy to answer any remaining concerns.

 Sincerely yours,

 Mihaly Koltai, on behalf of all authors

# Peer review: Reviewer response for version 1

**DOI:** 10.21956/wellcomeopenres.19063.r46312

**Published:** 2021-10-19

**Authors:** Zindoga Mukandavire

**Affiliations:** 1 Emirates Aviation University, Dubai, United Arab Emirates

The authors used a dynamic transmission model to analyse a time series of excess deaths in Mogadishu (Somalia) using data extracted from satellite images of six main cemeteries. The study aimed at estimating the probable date of introduction of SARS-CoV-2, the basic reproduction number and the effect of non-pharmaceutical interventions. This is a very interesting study which uses novel approaches to understand COVID-19 transmission dynamics in countries with weak surveillance systems or crisis-affected settings. I have the following comments.

1. Consider shortening the title of the paper to read “ *Date of introduction and transmissibility of severe acute respiratory syndrome coronavirus 2 (SARS-CoV-2) in Mogadishu, Somalia: estimates from mathematical modelling*”.
2. In the conclusion section of the abstract, it is not clear why estimates on transmissibility for Somalia are compared to European settings and not settings in Africa?
3. In data sources and statistical analysis section: Explain how you estimated that five smaller private and family-owned cemeteries accounted for < 20% of all burials. How did this affect the study findings?
4. How did the modelling results vary for seed sizes below 20?
5. The first column in Table 1, should be labelled “Parameter description”.

Is the work clearly and accurately presented and does it cite the current literature?

Yes

If applicable, is the statistical analysis and its interpretation appropriate?

Yes

Are all the source data underlying the results available to ensure full reproducibility?

Yes

Is the study design appropriate and is the work technically sound?

Yes

Are the conclusions drawn adequately supported by the results?

Yes

Are sufficient details of methods and analysis provided to allow replication by others?

Yes

Reviewer Expertise:

Applied Mathematics, Modelling Infectious Diseases

I confirm that I have read this submission and believe that I have an appropriate level of expertise to confirm that it is of an acceptable scientific standard.

# Response

**Published:** 2021-10-20

**Authors:** Mihaly Koltai

**Affiliations:** London School of Hygiene & Tropical Medicine, UK

Dear Dr. Mukandavire,

 Thank you for your review of our paper and the constructive suggestions.

 Responding to the points that you raised:

 1) We will consider shortening the title in a second version.

 2) Comparison was with European settings because more estimates were available for these. We will consider adding some more references on estimated R _0_ in African settings.

 3) This figure was based on the qualitative field work done by the colleagues at SDRI (Somali Disaster Resilience Institute) who verified the existence and functionality of burial sites. They concluded that these smaller private cemeteries accounted for a very small minority of burials. This conclusion was reached by considering the physical size of the cemeteries as well as by speaking to private individuals who were able to provide information on the burials within these sites (a limited number of prominent or very close family members were buried in these sites). When comparing this information with the information provided by the larger professionally managed cemeteries, our colleagues provided the <20% estimate. A more detailed description of data collection is now published in the adjoining study.

 4) Seed sizes smaller than 20 would push back the estimate for the date of introduction by another 5-14 days, depending on the exact value of assumed seed size. We think that date of introduction estimates even earlier than the ones presented are less likely than seed sizes of 20 or above.

 5) We can add the column name 'parameter description'.
